# Plasma Eicosapentaenoic Acid Is Associated with Muscle Strength and Muscle Damage after Strenuous Exercise

**DOI:** 10.3390/sports9010011

**Published:** 2021-01-14

**Authors:** Eisuke Ochi, Kenichi Yanagimoto, Yosuke Tsuchiya

**Affiliations:** 1 Faculty of Bioscience and Applied Chemistry, Hosei University, Tokyo 184-8584, Japan; 2 Graduate School of Sports and Health Studies, Hosei University, Tokyo 184-8584, Japan; 3 Food Function R&D Center, Nippon Suisan Kaisha, Ltd., Tokyo 105-8676, Japan; yanagimoto@nissui.co.jp; 4 Laboratory of Health and Sports Sciences, Meiji Gakuin University, Kanagawa 244-8539, Japan; yosuket@gen.meijigakuin.ac.jp

**Keywords:** omega–3 fatty acids, sports nutrition, ergogenic aid, long-chain *n* − 3 polyunsaturated fatty acids, lengthening, muscle function

## Abstract

Background: Although the ingestion of total omega–3 fatty acids (omega–3) is positively related with muscular strength in older persons, little is known about the effect of omega–3 plasma levels on muscular function before and after exercise in young men. Moreover, omega–3 supplementation has a positive role in exercise-induced acute muscle damage. This study investigated the relationship between plasma omega–3 in the blood and promotion and preservation of muscle strength after eccentric contractions (ECCs) in young men. Methods: Thirty-two healthy young men participated in this study. We assessed plasma omega–3 level and the maximal voluntary contraction (MVC). Twenty-six out of them exercised 60 ECCs at 100% MVC. We measured the MVC torque, flexibility before and immediately after exercise, 1–5 days post exercise. Results: The levels of eicosapentaenoic acid (EPA) and EPA/arachidonic acid were positively associated with muscle strength (*p* < 0.05). Higher levels of omega–3 EPA and docosahexaenoic acid prevented the reduction in the MVC and limited joint flexibility after ECCs. Conclusions: The present study reveals that higher levels of EPA are important to promote muscle strength and preserve the strength loss after exercise.

## 1. Introduction

Omega–3 fatty acids (omega–3) are the polyunsaturated fatty acid family of omega–3. Omega–3 fatty acids are included in fish oil and contain mainly eicosapentaenoic acid (EPA; 20:5 *n* − 3) and docosahexaenoic acid (DHA; 22:6 *n* − 3). Omega–3 was focused on because the Greenland Eskimos had a lower heart disease rate and consumed more fatty acids [1].

The types of muscular contraction are defined as isometric, shortening (concentric), or lengthening (eccentric; ECC) contractions. Especially ECC produces larger muscle force than other contractions, and causes greater muscle damage in untrained individuals [2,3]. It is well-known that ECC-induced muscular damage causes muscle strength loss, limited range of motion (ROM), delayed onset muscle soreness (DOMS), swelling, and serum creatine kinase (CK) and myoglobin (Mb) [2,3,4].

Some studies have studied the relationship between omega–3 intake and muscular function in older adults and young athletes [5,6,7]. Rossato et al. [6] reported that total omega–3 intake was positively correlated with knee strength in older males (50–85 years) but not in older females. In a study that examined the association between omega–3 blood levels and muscle function, total omega–3 was positively associated with knee extension strength [5]. These studies suggest that omega–3 consumption has a crucial role in maintaining muscle function in older males. However, Gravina et al. [7] reported that omega–3 supplementation did not cause any improvements in strength or power in young soccer players compared with placebo. Little is known about the relationship between the basal omega–3 levels in the blood and muscle function without the ingestion of supplementation in young males.

It is widely known that omega–3 effectively improves fatigue recovery and exercise performance [8,9]. In review papers, it is stated that omega–3 ingestion could be a new ergogenic aid for young adults and athletes [10,11]. In addition, we previously showed that 8-week EPA and DHA supplementation attenuated muscle damage following strenuous exercise [12,13]. This suggests that EPA and DHA could inhibit muscular damage after ECCs by protecting the membrane of muscle cells and reducing inflammatory response [8]. However, no investigation has studied the association between plasma omega–3 levels at baseline and ECCs-induced muscle damage without the ingestion of supplementation.

Accordingly, we examined the relationship between plasma omega–3 levels at baseline and muscle function, and muscle damage following ECCs without the ingestion of supplementation in young men. Collectively, the preliminary findings indicate that plasma omega–3 level is associated with muscle strength before and after exercise.

## 2. Materials and Methods

### 2.1. Subjects

Thirty-two healthy untrained men were included for this study (UMIN000016149, UMIN000024956, UMIN000028165, UMIN000033141). All subjects participated in our study. Twenty-six out of them agreed to participate in a study on the magnitude of muscle damage following ECCs. We requested to all subjects that they do not to attend other clinical interventions. No subject had done continuous exercise training within one year. All subjects were explained the whole study protocol before intervention and signed the informed consent form. The study was approved by the ethics committee for human experiments of Teikyo Heisei University (ID: R01–040) and followed the Declaration of Helsinki.

### 2.2. Study Design

Subjects visited the laboratory on one (6 subjects) or four (26 subjects) occasions throughout the study period. On the first visit, the blood samples were drawn from all subjects, and muscle strength of elbow flexion was assessed on the same day. After measurement of muscle strength, 26 subjects performed ECCs. All outcomes were obtained before and after ECCs, and on day 1, day 2, and day 5 after ECC at 9:00–11:00 a.m. in a temperature-controlled room maintained at 23 °C. According to previous studies [13,14,15,16], the ECCs and maximal voluntary contraction (MVC) tests were monitored and validated by a well-trained investigator (Y.T.).

### 2.3. Blood Sample

The subjects fasted for 8 h before a nurse took blood from the forearm. Then, the obtained blood was centrifuged for 10 min at 3k rpm at 4 °C [13]. The levels of blood plasma of fatty acids (EPA, DHA, and arachidonic acid (AA)) were analyzed.

### 2.4. Maximal Voluntary Contraction Torque

The MVC torque was calculated 3 times for 3-s contractions at 90° with a 1-min interval. Participants were encouraged to perform a maximal contraction during the tests. The mean value of the highest 1-s for each contraction was used for the MVC torque analysis. The detailed method of analysis is shown in our previous study [14].

### 2.5. ECCs

The participants sat on an arm curl bench. The weight of dumbbell was converted from MVC. The subjects performed 6 sets of a total of 10 ECCs of elbow flexors with a 1.5-min interval. The dumbbell was given to the participant with the elbow flexed at 90°, then made lower until it reached the extended position at 30°/s. A well-trained investigator returned the dumbbell so that the subject could restart the ECC.

### 2.6. Muscle Damage Markers

This study evaluated the MVC and range of motion (ROM) as muscle damage markers. The MVC is similar to the method of muscle strength measurement described above. The investigator measured the extended and flexed arm to calculate the elbow ROM as shown in a previous study [14].

### 2.7. Statistical Analyses

Statistical analysis was conducted with SPSS software version 26.0 (IBM Corp., Armonk, NY, USA). Values were expressed as means ± standard deviation. The physiological characteristics of subjects and muscle strength were generated for participants by quartiles of EPA, DHA, and EPA/AA in order to investigate whether blood EPA and DHA level affect muscle function based on previous studies [6,17]. A 1-way ANOVA was conducted on the quartiles based on a previous study [2]. In addition, Pearson’s correlation coefficient was used to assess the association between muscle strength and EPA, DHA, EPA per AA. Muscle damage markers were generated for participants by tertiles of EPA, DHA, EPA per AA. The ECC-induced muscle damage was performed with 2-way ANOVA. Bonferroni’s correction was used to following post-hoc testing. We considered *p* < 0.05 to be significant.

## 3. Results

The subject physical characteristics and range of concentration in the plasma EPA, DHA, EPA per AA by the Quartile can be seen in Table 1. As for body mass, Quartile 4 of EPA was higher than Quartile 1 and 2 (*p* < 0.05). Furthermore, Quartile 4 of DHA was higher than Quartile 2 (*p* < 0.05). Moreover, as for BMI, Quartile 4 of EPA was higher than Quartile 1 (*p* < 0.05), and Quartile 4 of DHA was higher than Quartile 1 and 2 (*p* < 0.05). In addition, we have analyzed anthropometric data by Tertiles 1, 2, and 3 and the results are similar to Quartile 4. The range of concentration were as follows; EPA: Tertiles 1, 9.4–15.7 μg/mL, Tertiles 2, 17.3–25.6 μg/mL, and Tertiles 3, 26.0–94.5 μg/mL, DHA: Tertiles 1, 35.4–58.4 μg/mL, Tertiles 2, 58.4–78.2 μg/mL, and Tertiles 3, 82.9–119.2 μg/mL, EPA/AA: Tertiles 0.06–0.09, Tertiles 2, 0.09–0.1, and Tertiles 3, 0.13–0.5. For body mass, Tertile 3 of EPA (71.2 ± 6.7 kg), DHA (70.6 ± 6.6 kg), and EPA/AA (68.1 ± 6.1 kg) were significantly higher than that of Tertile 1 (EPA: 60.2 ± 6.5 kg, DHA: 62.4 ± 8.4 kg, EPA/AA: 60.2 ± 7.2 kg, *p* < 0.05). For BMI, Tertile 3 of EPA (24.2 ± 2.2) and DHA (23.9 ± 2.2) were significantly higher than that of Tertile 1 (EPA: 20.6 ± 2.5, DHA: 20.9 ± 2.6, *p* < 0.05).

**Figure 1:**
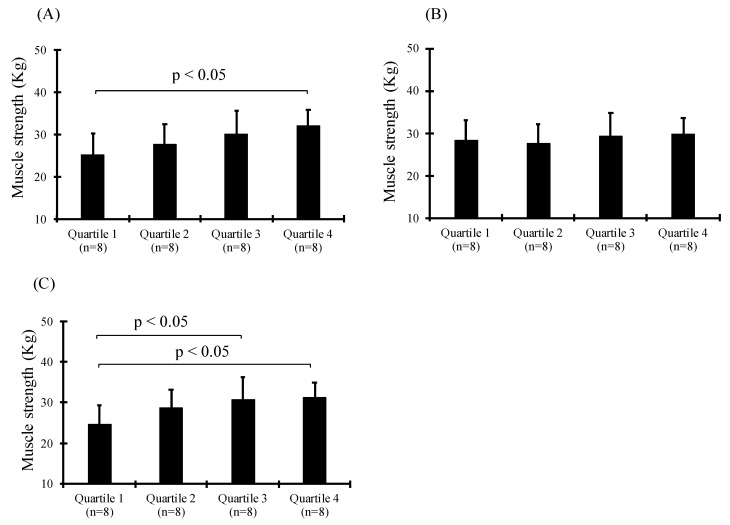
Comparison of muscle strength in the blood levels of EPA (**A**), DHA (**B**), and EPA/AA (**C**).

**Figure 2:**
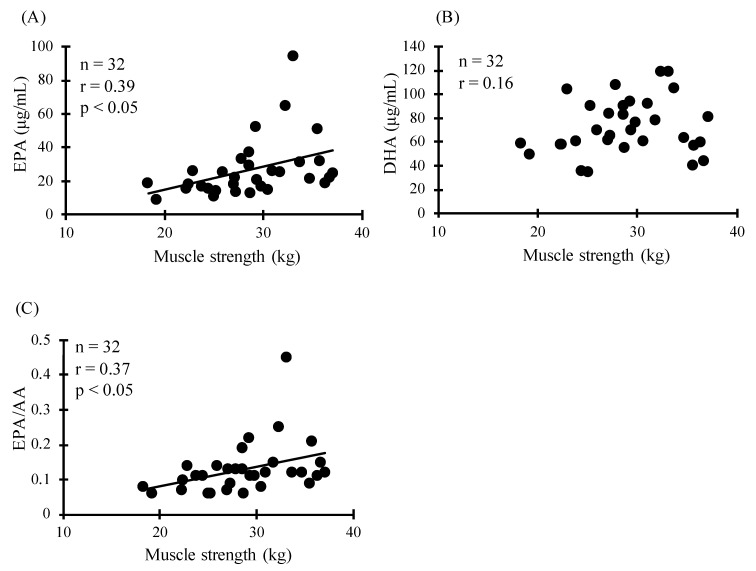
Relationship between the blood level of muscle strength and EPA (**A**), DHA (**B**), and EPA/AA (**C**).

As for muscle strength, Quartile 4 of EPA was significantly higher than that of Quartile 1 (Figure 1A, *p* < 0.05). In addition, Quartile 4 and 3 of EPA/AA were higher than Quartile 1 (Figure 1C, *p* < 0.05). However, there was no association between DHA and muscle strength (Figure 1B). This was corroborated by the correlation between muscle strength and plasma EPA, and plasma EPA/AA (Figure 2, *p* < 0.05).

**Figure 3:**
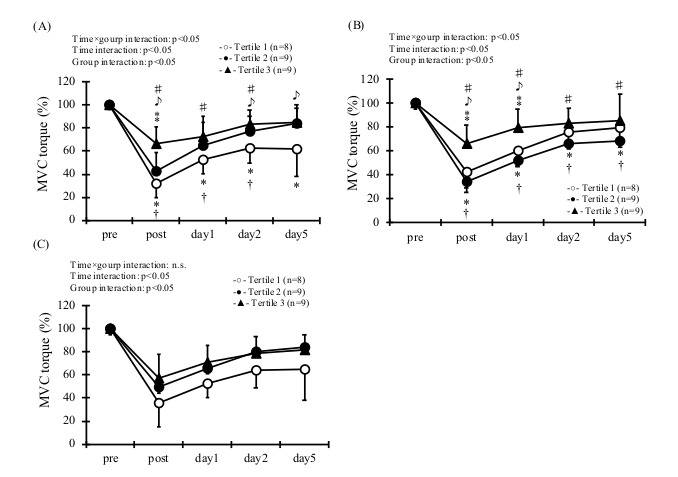
Maximal voluntary contraction (MVC) at pre and post, and then 1, 2 and 5 days in the blood level of EPA (**A**), DHA (**B**), and EPA/AA (**C**). * *p* < 0.05, from pre- in the Tertile 1. † *p* < 0.05, from pre- in the Tertile 2. ♯ *p* < 0.05, from pre- in the Tertile 3. ♪ *p* < 0.05, between Tertile 1 and 3. ⁑ *p* < 0.05, between Tertile 2 and 3.

**Figure 4:**
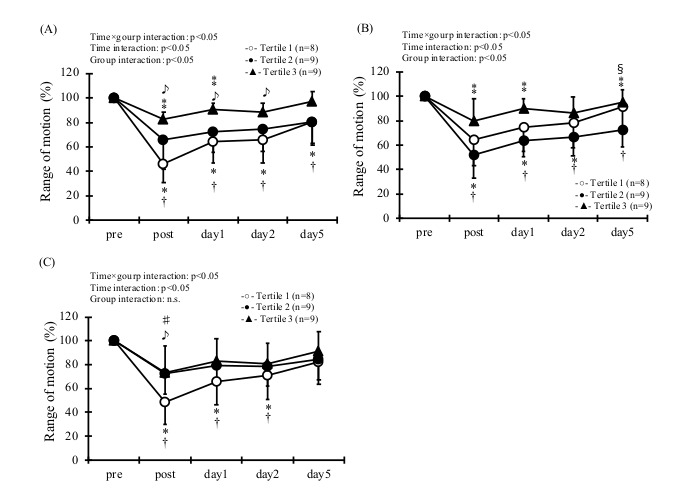
Range of motion ROM at pre and post, and then 1, 2 and 5 days in the blood level of EPA (**A**), DHA (**B**), and EPA/AA (**C**). * *p* < 0.05, from pre- in the Tertile 1. † *p* < 0.05, from pre- in the Tertile 2. ♯ *p* < 0.05, pre- in the Tertile 3. ♪ *p* < 0.05, between Tertile 1 and 3. ⁑ *p* < 0.05, between Tertile 2 and 3. § *p* < 0.05, between Tertile 1 and 2.

Regarding the association between omega–3 and ECC-induced muscle damage, statistical analysis showed a group × time interaction effect for the MVC in EPA and DHA (Figure 3, *p* < 0.05). As for MVC, Tertile 3 of EPA was higher than Tertile 1 and 2 at post-exercise (*p* < 0.05). In addition, Tertile 3 of EPA was higher than Tertile 1 at 2 d and 5 d post exercise (*p* < 0.05). Tertile 3 of DHA was significantly higher than that of Tertile 1 and 2 at post- and 1 d post-exercise (*p* < 0.05). In addition, there was a group × time effect for ROM in EPA, DHA, and EPA/AA (Figure 4, *p* < 0.05). As for ROM, Tertile 3 of EPA was significantly higher than that of Tertile 1 and 2 at post- and 1 d post-exercise (*p* < 0.05). Moreover, Tertile 3 of EPA was higher than Tertile 1 at 2 d post-exercise (*p* < 0.05). Similarly, Tertile 3 of DHA was significantly higher than that of Tertile 1 and 2 immediately after exercise and 1 d, 5 d post-exercise (*p* < 0.05). Moreover, Tertile 3 of EPA/AA was significantly higher than that of Tertile 1 immediately after exercise (*p* < 0.05).

## 4. Discussion

This study investigated the association between the level of EPA, DHA, and EPA per AA in the blood and the promotion of muscle strength and preservation of strength loss after ECCs in young men. In addition, we also investigated the effect of EPA, DHA, EPA per AA on ECCs-induced muscle damage. The main findings of our study were (1) the concentrations of EPA and EPA per AA were correlated with muscular function in healthy young males; (2) higher levels of EPA, DHA, EPA per AA have preventive roles for preserving muscle strength and joint flexibility after strenuous exercise in healthy young men. Therefore, higher plasma omega–3 is positively associated with muscle strength in young men, and that preserves muscle damage following exercise. To the best of our knowledge, this is the first study to show their relationship.

This study showed that higher EPA, DHA, and EPA per AA resulted in higher body weight and BMI. A previous study reported that higher consumption of omega–3 showed higher body mass, higher lean mass, and lower body fat [6]. In addition, Reinders et al. [5] showed that higher plasma concentrations of omega–3 were associated with larger thigh cross-sectional muscle area in older adults. Although we did not measure body composition (lean mass and body fat), we speculate that plasma levels of EPA and DHA may be related to body composition, especially muscle mass in young men.

Omega–3 supplementation enhances the rate of muscle protein synthesis in older adults [18], and improves in muscle strength and functional capacity after strength training in older women [19]. In addition, Rossato et al. [6] showed that the intake of omega–3 was positively associated with muscle strength in older men, but not in older women. Considering the results of previous studies and this study, omega–3 blood levels, especially EPA, are positively associated with muscle strength in young men.

The present study also reveals that higher EPA and DHA levels are important to reduce muscle damage. Our previous study showed that 8–week omega–3 supplementation caused inhibition in decreased muscle strength, reduced ROM, muscle soreness [13]. In addition, previous studies also demonstrated that omega–3 or arachidonic acid are associated with nerve conduction [12,20]. Hence, the neurological effect may be involved in the cause of omega–3 level reducing muscle damage following ECCs.

This study has several limitations. The subjects were limited to young men, and the sample size was not enough to reach a conclusion on our hypothesis. Further studies are needed to compare elderly participants with young ones in a large sample. Moreover, the only muscle damage markers evaluated muscle strength and ROM in this study. It is necessary to study the EPA and DHA levels on another symptom of muscular damage such as plasma CK, Mb, and inflammatory response.

## 5. Conclusions

We conclude that higher plasma omega–3 is positively related with muscular function in young males. Furthermore, higher blood levels of EPA and DHA preserve muscle and joint function following exercise. Therefore, although the present study shows preliminary data, we propose a daily intake of omega–3 might be important to maintain to promote and preserve the muscle strength after exercise.

## Figures and Tables

**Table 1:** Physical characteristics and rage of concentration in the EPA, DHA, and EPA/AA.

	Quartile	Rage of Concentration (μg/mL)	Age (y)	Height (cm)	Body Mass (kg)	BMI
EPA	Quartile 1 (*n* = 8)	6.9–15.7	19.9 ± 1.0	170.4 ± 8.9	60.2 ± 7.7	20.8 ± 2.9
	Quartile 2 (*n* = 8)	17.3–22.3	20.0 ± 1.2	171.6 ± 3.9	63.9 ± 7.4	21.7 ± 2.2
	Quartile 3 (*n* = 8)	22.4–29.7	20.4 ± 1.1	171.7 ± 6.6	65.2 ± 6.0	22.2 ± 2.3
	Quartile 4 (*n* = 8)	30.4–94.5	20.0 ± 1.1	173.5 ± 7.2	73.0 ± 5.3 *†	24.3 ± 1.8 *
DHA	Quartile 1 (*n* = 8)	35.4–58.4	19.8 ± 1.0	173.3 ± 8.7	63.5 ± 9.0	21.2 ± 3.0
	Quartile 2 (*n* = 8)	58.5–66.6	19.9 ± 1.1	170.0 ± 6.1	60.7 ± 6.1	21.0 ± 1.5
	Quartile 3 (*n* = 8)	70.0–83.7	20.3 ± 0.9	171.2 ± 5.1	65.7 ± 6.6	22.4 ± 2.2
	Quartile 4 (*n* = 8)	90.8–119.2	20.4 ± 1.2	172.6 ± 7.0	72.6 ± 5.1 †	24.4 ± 2.1 *†
EPA/AA	Quartile 1 (*n* = 8)	0.05–0.08	19.9 ± 1.0	168.4 ± 7.4	60.7 ± 8.6	21.4 ± 3.4
	Quartile 2 (*n* = 8)	0.09–0.12	20.0 ± 1.2	174.3 ± 5.8	65.8 ± 9.0	21.6 ± 2.3
	Quartile 3 (*n* = 8)	0.12–0.14	20.8 ± 0.9	170.4 ± 4.2	69.2 ± 7.3	23.8 ± 2.3
	Quartile 4 (*n* = 8)	0.20–0.50	19.6 ± 0.9	174.1 ± 7.8	68.9 ± 5.8	22.8 ± 1.9
All subjects (*n* = 32)		―	20.1 ± 1.0	171.8 ± 6.6	65.6 ± 7.9	22.2 ± 2.6

* *p* < 0.05 versus. Quartile 1, † *p* < 0.05 versus Quartile 2.

## Data Availability

The data presented in this study are available on request from the corresponding author.
